# FOD Detection Method Based on Iterative Adaptive Approach for Millimeter-Wave Radar

**DOI:** 10.3390/s21041241

**Published:** 2021-02-10

**Authors:** Yangliang Wan, Xingdong Liang, Xiangxi Bu, Yunlong Liu

**Affiliations:** 1 National Key Lab of Microwave Imaging Technology, Aerospace Information Research Institute, Chinese Academy of Sciences, Beijing 100190, China; ylwan@mail.ie.ac.cn (Y.W.); buxiangxi14@mails.ucas.edu.cn (X.B.); liuyl003299@aircas.ac.cn (Y.L.); 2 School of Electronic, Electrical and Communication Engineering, University of Chinese Academy of Sciences, Beijing 100049, China

**Keywords:** foreign object debris (FOD), millimeter-wave radar, iterative adaptive approach (IAA), interference suppression, false alarms reduction, super-resolution

## Abstract

Using millimeter-wave radar to scan and detect small foreign object debris (FOD) on an airport runway surface is a popular solution in civil aviation safety. Since it is impossible to completely eliminate the interference reflections arising from strongly scattering targets or non-homogeneous clutter after clutter cancellation processing, the consequent high false alarm probability has become a key problem to be solved. In this article, we propose a new FOD detection method for interference suppression and false alarm reduction based on an iterative adaptive approach (IAA) algorithm, which is a non-parametric, weighted least squares-based iterative adaptive processing approach that can provide super-resolution capability. Specifically, we first obtain coarse FOD target information by data preprocessing in a conventional detection method. Then, a refined data processing step is conducted based on the IAA algorithm in the azimuth direction. Finally, multiple pieces of information from the two steps above are used to comprehensively distinguish false alarms by fusion processing; thus, we can acquire accurate FOD target information. Real airport data measured by a 93 GHz radar are used to validate the proposed method. Experimental results of the test scene, which include golf balls with a diameter of 43 mm, were placed about 300 m away from radar, which show that the proposed method can effectively reduce the number of false alarms when compared with a traditional FOD detection method. Although metal balls with a diameter of 50 mm were placed about 660 m away from radar, they also can obtain up to 2.2 times azimuth super-resolution capability.

## 1. Introduction

Foreign object debris (FOD) can severely injure airport or airline personnel or damage equipment [1]. Considering the huge losses caused by FOD, radar-based FOD detection deserves more attention, due to its many advantages at all times and under all weather conditions [2,3,4]. Compared with infrared, laser, TV, and other optical sensors, millimeter-wave radar has a stronger ability to penetrate fog, smoke, and dust, while having the characteristics of high spatial resolution [5,6,7,8]. Therefore, millimeter-wave radar techniques for detecting FOD have become a research hot spot.

When a millimeter-wave radar scans an entire runway, the received echo not only contains the reflection information of the target, but also contains clutter and interference signals generated by the ground, nearby buildings, airplanes, and cars beside the runway. A commonly used method is the constant false alarm rate (CFAR) detection algorithm. There are two common methods for implementing a constant false alarm rate (CFAR) processor [9]. In one, the detector outputs of nearby cells (range and/or azimuth) are averaged to obtain a background estimate, which is used for thresholding in the space domain. These methods include cell-average CFAR (CA-CFAR) [10,11], the greatest or smallest option (GO or SO) [12], and ordered statistic (OS) [13] techniques. When the clutter background becomes complicated or the target is in a non-homogeneous clutter environment, the detection performance of these methods may be greatly degraded [14]. In the other type of method, the detector output of each resolution cell is averaged over several scans, in order to obtain the background estimate—namely, the clutter-map (CM)—in the time domain. Although many improved algorithms have been proposed, they still have certain performance limitations [15,16,17,18]. Meanwhile, some hybrid methods are proposed for FOD detection in W band radar. A method of trimmed-mean clutter-map CFAR detection based on OS is proposed in multiple target environments [19]. In [20], the authors presented a threshold-improved approach based on the cell-averaging clutter-map (CA-CM-) CFAR. The clutter map Constant False Alarm Rate (CFAR) detection algorithm is utilized firstly to categorize radar echoes into two kinds, then a novel hierarchical FOD detection method is proposed based on feature extraction and support vector domain description [21,22,23,24]. Due to the clutter and interference, the detection results of CFAR and improved-CFAR methods are often simultaneously accompanied by many false alarms. The high false alarm probability of weak target detection under strong complex and non-homogeneous clutter background remains a key problem.

Recently, the iterative adaptive approach (IAA) [25], which is a non-parametric, weighted least squares-based iterative adaptive processing approach, has been adopted for high-resolution scanning radar images, in order to estimate signal amplitude and phase [26,27,28,29]. IAA is a super-resolution algorithm which offers superior interference and noise suppression performance when few snapshots are available and the signal-to-noise ratio (SNR) is low [30,31,32,33,34]. Based on the above analysis, we introduce an IAA technique for the signal processing of FOD targets, suppressing the dispersive and residual interference which comes from strongly scattering targets or non-homogeneous clutter after clutter cancellation processing. This method uses the adaptive iteration of the IAA algorithm to estimate the part of the clutter component during each iteration and eliminate its influence from the measured data, thereby achieving interference suppression. To verify the practical effect of this method, a W-band linear frequency modulation continuous wave radar was used to collect data at a general aviation airport in Beijing, China. Based on the acquired data, we compared the proposed method with a traditional FOD detection method. The proposed method can effectively eliminate the false targets caused by strong target side-lobe interference and the single discrete clutter component after clutter cancellation processing, while the clutter background is simultaneously smoothed. Overlapping targets in the same range bin can also be distinguished effectively, and the azimuth resolution is improved. Therefore, the method proposed in this paper can actually be used to improve the detection of foreign object debris on airport runways.

This paper is organized as follows: The geometric and signal models of the real aperture FOD scanning radar are described in Section 2. In Section 3, the detection method based on IAA is derived in detail. Section 4 describes the experimental scenarios. The processed results of real data and the discussion are presented in Section 5. The conclusions and final discussion are drawn in Section 6.

## 2. Models

**Figure 1:**
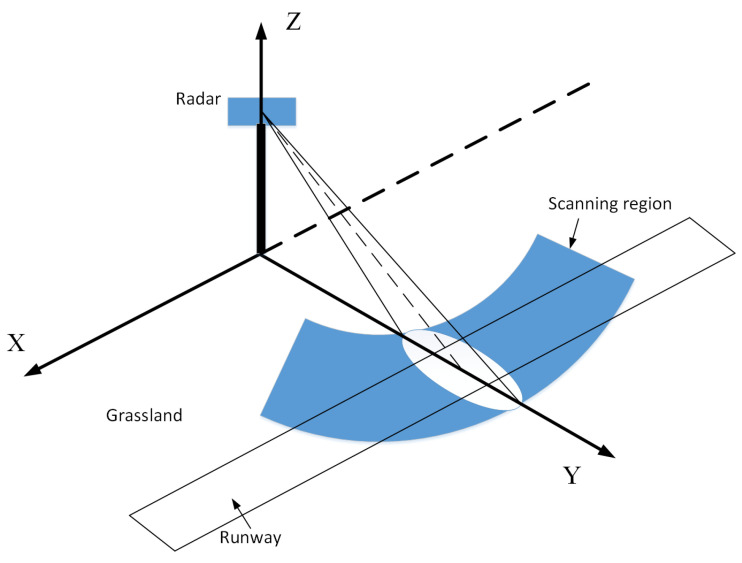
Geometry of FOD millimeter-wave radar illumination.

An FOD millimeter-wave detection radar is generally deployed on both sides of the runway, where the radar antenna scans and illuminates the rough runway surface. The geometric model of FOD millimeter-wave radar illumination is shown in Figure 1.

The radar needs to detect small foreign objects within a short distance, which requires the radar to have the smallest possible distance blind zone and higher range resolution. A millimeter-wave radar usually uses the linear frequency modulation continuous wave system, assuming that the transmitted signal is

(1) $$s\left(\tau \right)=exp\left[j2\pi \left(f_{0}\tau +\frac{1}{2}u\tau^{2}\right)\right],-\frac{T}{2}\leq \tau \leq \frac{T}{2},$$

where τ denotes the fast time, $f_{0}$ is the center frequency of the signal, u=B/T is the frequency modulation rate of the chirp signal, *B* is frequency sweep bandwidth, *T* is frequency sweep cycle, and the target echo signals at different distances can be expressed as

(2) $$s_{R}\left(\tau \right)=exp\left[j2\pi \left(f_{0}\left(\tau -\tau_{d}\right)+\frac{1}{2}u{\left(\tau -\tau_{d}\right)}^{2}\right)\right],-\frac{T}{2}\leq \tau \leq \frac{T}{2},$$

where $\tau_{d}=2R/c$ is the echo delay caused by the target, *R* is the target slant range, and *c* is the velocity of light. The difference frequency signal can be obtained after mixing and filtering the echo signal

(3) $$s_{b}\left(\tau \right)=s\left(\tau \right)\times s_{R}^{*}\left(\tau \right)=exp\left\{j2\pi \left[u\frac{2R}{c}\tau -u\frac{2R^{2}}{c^{2}}+\frac{2R}{\lambda }\right]\right\},-\frac{T}{2}\leq \tau \leq \frac{T}{2},$$

where $s_{R}^{*}\left(\tau \right)$ is the conjugate of $s_{R}\left(\tau \right)$, Equation (3) is the “matched filter” processing of the LFMCW. This signal is a single-frequency signal with a frequency of $f_{1}=u\frac{2R}{c}$, where λ is the radar wavelength. After Fast Fourier Transform (FFT) processing, the range echo can be expressed as

(4) $$S_{b}\left(f\right)=Tsinc\left[T\left(f-f_{1}\right)\right]expj2\pi [-u\frac{2R^{2}}{c^{2}}+\frac{2R}{\lambda }].$$

Performing the time–frequency substitution of f→τ yields

(5) $$S_{b}\left(\tau \right)=Tsinc\left[T\left(u\tau -u\frac{2R}{c}\right)\right]expj2\pi [-u\frac{2R^{2}}{c^{2}}+\frac{2R}{\lambda }]=Tsinc\left[B\left(\tau -\frac{2R}{c}\right)\right]expj2\pi [-u\frac{2R^{2}}{c^{2}}+\frac{2R}{\lambda }].$$

After the range “matched filter” and Fast Fourier Transform (FFT) processing (we call it “range compression”), the range-azimuth two-dimensional echo signal can be expressed as

(6) $$g\left(t,\tau \right)=\sum_{j=1}^{M}\sum_{i=1}^{N}\sigma_{ij}h\left(t\right)sinc\left[B\left(\tau -\frac{2R}{c}\right)\right]exp\left[j2\pi \left(-u\frac{2R^{2}}{c^{2}}+\frac{2R}{\lambda }\right)\right],$$

where *t* is the slow time, *N* is the number of range sampling points, and *M* is the number of azimuth sampling points, $\sigma_{ij}$ means the scatting amplitude of target located at *i*th range and *j*th azimuth bin, and h(t) is the antenna pattern modulation function.

**Figure 2:**
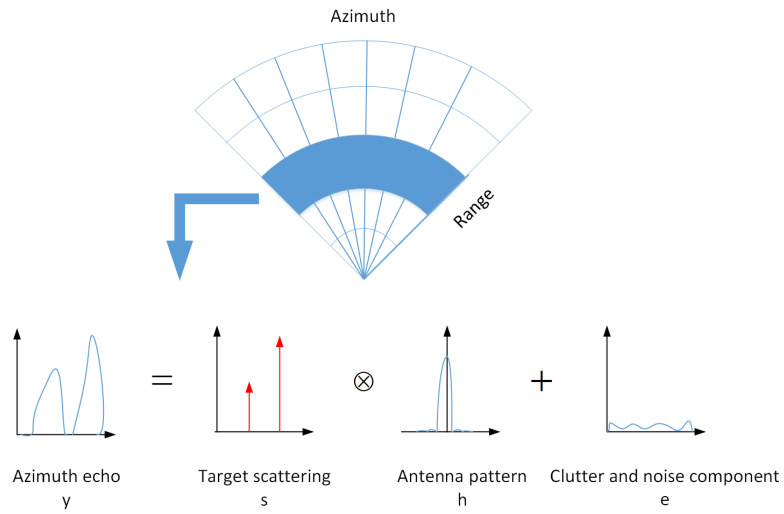
Signal model of the target azimuth echo for the scanning FOD millimeter-wave radar.

In scanning radar surface mapping, the azimuth echo can be regarded as the convolution result between the antenna pattern and the reflectivity function. The recorded echo data by radar is inevitably mixed with ground clutter and noise components, although clutter cancellation processing is performed before target detection. A signal model of the target azimuth echo for the scanning FOD millimeter-wave radar is shown in Figure 2. For each range unit, the discrete signal model of the target azimuth echo $y\in C^{M\times 1}$ can be expressed as

(7) y=s⊗h+e,

where ⊗ is the convolution operation, *s* is the target scattering distribution, *h* is the antenna pattern, and *e* is the discrete clutter and additive noise component after clutter cancellation. This formula is equivalent to

(8) $$y=\left.\left(\begin{array}{cccc} h_{1} & & & \\ h_{2} & h_{1} & & \\ \vdots & h_{2} & \ddots & \\ h_{L} & \vdots & \ddots & h_{1}\\ \; & h_{L} & \; & h_{2}\\ \; & \; & \ddots & \vdots \\ \; & \; & & h_{L} \end{array}\right)\right.s+e,$$

where *s* is the target scattering vector defined as $s\overset{▵}{=}{\left(s_{1},s_{2},\cdots s_{K}\right)}^{T}$, suppose that there are *K* point targets, $h\overset{▵}{=}{\left(h_{1},h_{2},\cdots h_{L}\right)}^{T}$ defines the antenna pattern vector, where ${\left(.\right)}^{T}$ represents transposition, and *L* is the number of antenna pattern sampling points. Furthermore, the steering vector matrix *A* can be defined as

(9) $$A=\left.\left(\begin{array}{cccc} h_{1} & & & \\ h_{2} & h_{1} & & \\ \vdots & h_{2} & \ddots & \\ h_{L} & \vdots & \ddots & h_{1}\\ \; & h_{L} & \; & h_{2}\\ \; & \; & \ddots & \vdots \\ \; & \; & & h_{L} \end{array}\right)\right.\overset{▵}{=}\left(a_{1},a_{2},\cdots a_{K}\right),$$

where $h_{l}\neq 0,l=1,\ldots ,L$. The relationship among *M*, *K*, and *L* can be determined by

(10) M=K+L−1.

## 3. Detection Method

### 3.1. Interference Suppression and Super-Resolution Based on IAA

The iterative adaptive approach (IAA) is a non-parametric, weighted least squares-based iterative adaptive spectrum estimation approach. It can provide high resolution and low side-lobe levels in the case of a small number of snapshots, coherent sources, and low SNR [35]. Due to these advantages, the IAA has been applied to scanning radar sensing and demonstrated as outstanding, in terms of both resolution improvement and noise suppression. In the IAA framework [36], Equation (8) can be solved by weighted least squares (WLS) minimization. The WLS cost function is given by

(11) $${\left[y-s_{k}a_{k}\right]}^{H}Q^{-1}\left(k\right)\left[y-s_{k}a_{k}\right],k=1,2,\ldots K,$$

where ${\left(.\right)}^{H}$ represents conjugate transpose, Q(k) is the interference (signals at angles other than the angle of current interest *k*) covariance matrix

(12) $$Q\left(k\right)=\hat{R}-\hat{P_{k}}a_{k}a_{k}^{H}.$$

Define the covariance matrix of the echo $\hat{R}\overset{▵}{=}APA^{H}$ and let P be a K×K diagonal matrix, whose diagonal contains the power at each angle on the scanning grid. Then, *P* can be expressed as

(13) $$\hat{P_{k}}={\left|\hat{s_{k}}\right|}^{2},k=1,2,\ldots ,K.$$

Minimizing the cost function (10) with respect to $s_{k}$ yields

(14) $$\hat{s_{k}}=\frac{a_{k}^{H}{Q(k)}^{-1}y}{a_{k}^{H}{Q(k)}^{-1}a_{k}}.$$

It can be seen, from the above formula, that a huge computational burden is required to calculate the value of $s_{k}$, as recalculating Q(k) is required in each step. Fortunately, by the matrix inversion lemma, $Q{\left(k\right)}^{-1}$ can be expressed as

(15) $$Q{\left(k\right)}^{-1}={\hat{R}}^{-1}+\frac{\hat{P_{k}}{\hat{R}}^{-1}a_{k}a_{k}^{H}{\hat{R}}^{-1}}{1-\hat{P_{k}}a_{k}^{H}{\hat{R}}^{-1}a_{k}}.$$

Inserting (15) into (14), the iterative formula for WLS estimation can be obtained as:

(16) $$\hat{s_{k}}=\frac{a_{k}^{H}{\hat{R}}^{-1}y}{a_{k}^{H}{\hat{R}}^{-1}a_{k}}.$$

The azimuth echo is constructed by convolution of the antenna pattern and the target scattering coefficient, including the presence of noise. The signal recovery process is transformed into the corresponding inversion process. As the iteration proceeds, the IAA covariance matrix $\hat{R}$ approaches a singular matrix and, so, its ill conditioning is inevitable. By adding regular terms, introducing prior information of noise or clutter distribution, and restoring the rank, the ill-conditioned problem can be handled. Some white noise is artificially added to the main diagonal of the echo data covariance matrix; namely, adaptive diagonal loading technology. This can ensure that the diagonal loading matrix is always invertible, regardless of whether the covariance matrix is singular or not. The covariance matrix of echo data can be expressed as

(17) $$\hat{R}=A\hat{P}A^{H}+\hat{\xi }I,$$

where $\hat{\xi }$ is the diagonal loading factor and *I* is an identity matrix. According to the regularized IAA algorithm [26], the optimized parameters can be calculated by the following formula:

(18) $$\hat{\xi }=\frac{1}{M}\sum_{m=1}^{M}{\left|\frac{i_{m}^{H}{\hat{R}}^{-1}y}{i_{m}^{H}{\hat{R}}^{-1}i_{m}}\right|}^{2},$$

where *M* is the length of echo data and $i_{m}$ is the mth column of the identity matrix *I*.

The above-mentioned dispersive and residual interference mainly comes from the side-lobe of strongly scattering targets or non-homogeneous clutter after clutter cancellation processing. We use the adaptive iteration of the IAA algorithm to estimate the part of the interference component during each iteration and eliminate its influence from the measured data, thereby achieving interference suppression. The interference suppression and super-resolution algorithm based on IAA is implemented in an iterative manner, as described in Algorithm 1. Usually, the initialization is done by a standard delay-and-sum beamformer [35] that is, $P_{k}=\frac{1}{{\left(a_{k}^{H}a_{k}\right)}^{2}}{\left|a_{k}^{H}y\right|}^{2},k=1,2,\ldots ,K$. When the difference between two adjacent estimated powers is calculated to be less than a certain threshold or a prescribed number of iterations is reached, the estimate of $\hat{s}$ is obtained.
**Algorithm 1** Interference suppression and super-resolution algorithm based on IAA**Initialization**: $P_{k}=\frac{1}{{\left(a_{k}^{H}a_{k}\right)}^{2}}{\left|a_{k}^{H}y\right|}^{2},k=1,2,\ldots ,K$**Repeat**$\hat{R}=A\hat{P}A^{H}+\hat{\xi }I$**for***k* = 1, 2, …, *K*$\hat{s_{k}}=\frac{a_{k}^{H}{\hat{R}}^{-1}y}{a_{k}^{H}{\hat{R}}^{-1}a_{k}}$$\hat{P_{k}}={\left|\hat{s_{k}}\right|}^{2}$$\hat{\xi }=\frac{1}{M}\sum_{m=1}^{M}{\left|\frac{i_{m}^{H}{\hat{R}}^{-1}y}{i_{m}^{H}{\hat{R}}^{-1}i_{m}}\right|}^{2}$**end for****until** (convergence)

### 3.2. FOD Detection Method

Traditional FOD detection methods only use the background clutter to cancel the clutter of the observed scene, which makes it impossible to completely eliminate the clutter and interference reflections. The proposed method combines the clutter cancellation together, while exploiting the sparsity of the observed scene and distribution characteristics of interference. The dispersed interference reflections can be suppressed under the process of IAA reconstruction.

Based on the existing FOD detection algorithm, we propose an FOD detection method based on IAA signal reconstruction. The method is divided into three steps: data preprocessing, refined data processing, and information fusion processing. Finally, accurate FOD target information (coordinate information on the runway) can be obtained.

Specific descriptions of the steps are as follows:

**Step 1:**  Data preprocessing. First, the estimation of clutter background intensity is obtained by averaging the measured values of previous multiple scans of the runway scene without targets. After iterative averaging, the clutter map storage value becomes more and more stable, and the clutter change amplitude becomes smaller and smaller, which can reduce the false alarm rate. The adaptive clutter map CFAR technology is used to obtain the coarse FOD target information. This is the general detection method of conventional FOD radar.
**Step 2:**  Refined data processing. First, use standard instruments to acquire the radar antenna pattern data by far field measurement method, which can be normalized for subsequent processing. Then, according to the coarse FOD target information obtained in Step 1, the original data of the same range bin corresponding to the FOD target position will be reprocessed by IAA in the azimuth direction. Finally, the CFAR detection processing is performed again.
**Step 3:**  Information fusion processing.Through the processing of the above two steps, the coarse FOD target information obtained by Step 1 and the second detection FOD target information acquired by Step 2 are used to comprehensively distinguish false alarms by fusion processing of multiple information, in order to obtain accurate FOD target information.

**Figure 3:**
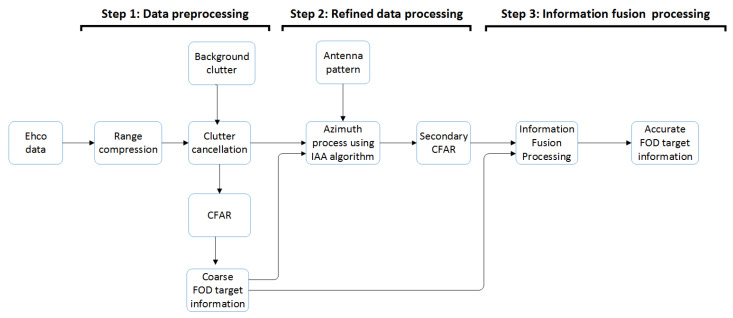
Flowchart of the FOD radar processing chain for the proposed method.

The specific flow chart is shown in Figure 3. This article focuses on the IAA processing part of Step 2.

## 4. Experimental Scenarios

To validate the effectiveness of the method proposed in this paper, some experimental results using FOD millimeter-wave radar data from the Aerospace Information Research, Chinese Academy of Sciences (AIRCAS) are presented. We performed our experiment using real Airport data. In the following, we introduce the radar sensor and test scenarios.

### 4.1. Radar Sensor

**Figure 4:**
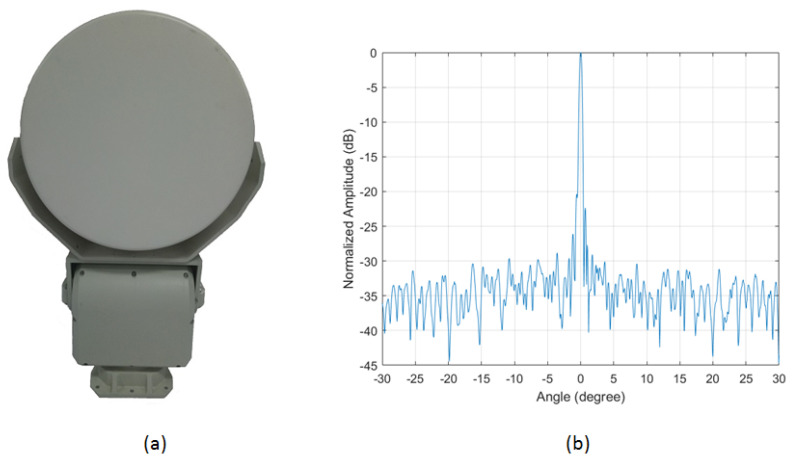
Some details of FOD millimeter-wave radar: (**a**) radar appearance; and (**b**) measured antenna azimuth radiation pattern at 93 GHz.

The test radar was a W-band linear frequency modulation continuous wave radar with a repetition frequency of 1000 Hz. The radio-frequency (RF) signals with a bandwidth of 2 GHz from 92 GHz to 94 GHz were downsampled into an intermediate-frequency (IF) band and loaded via an AD converter with sampling frequency of 50 MHz. The radar was mounted on a mechanical servo motor, in order to achieve azimuth scanning. A photo of the radar is shown in Figure 4a.

The antenna adopted a pair of horizontally polarization-fed parabolic antennae and scanned horizontally across an angular range of 360 degrees (according to this test scenario, we set the scan angle range from −40 degrees to +70 degrees). The basic function of the antenna is to complete signal transmission and reception. The transceiver antenna was separated to improve the isolation. According to the performance requirements of the system, an offset-fed parabolic antenna was used, with the transceiver antenna having the same form. The main-lobe beamwidth of the antenna was 4 degrees in elevation and 0.6 degree in azimuth. The narrow azimuthal beamwidth and wide elevation beamwidth make this antenna practical for FOD detection. Figure 4b shows the measured azimuth radiation pattern of the antenna at 93 GHz. The working parameters of the FOD millimeter-wave radar system are given in Table 1.

### 4.2. Test Scenarios

**Figure 5:**
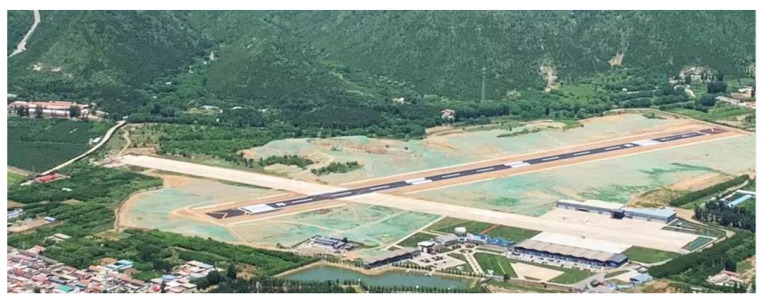
Photo of Beijing Miyun Airport.

We performed our experiment at Beijing Miyun Airport, located in the northeast of the Beijing Miyun District. The new airport runway (asphalt material) and the crossed old runway (concrete material) are both 800 m in length. Figure 5 shows a photo of the airport.

**Figure 6:**
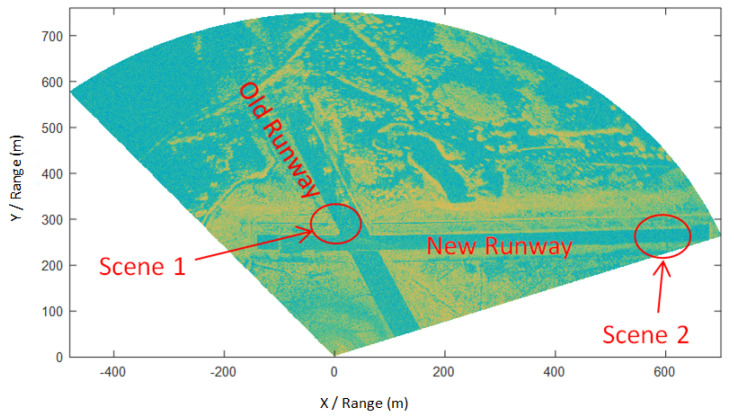
Radar amplitude image of Beijing Miyun Airport.

The test radar was placed about 250 m from the center line of the new runway, with the radar antenna about 9 m above the ground—after clearing the runway and manually confirming that there were no FODs in the test scene. The original echo data of the entire airport was acquired by scanning the airport. After range compression processing, the center of the radar antenna was used as the co-ordinate origin. Then, a radar amplitude image of the entire airport could be obtained, which can be used as the background clutter for clutter cancellation processing, as shown in Figure 6.

**Figure 7:**
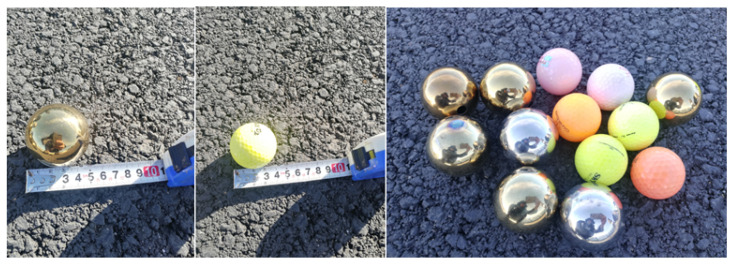
FOD targets used in the experiment.

In order to verify the interference suppression and super-resolution improvement of FOD radar detection by the proposed IAA algorithm, we selected two test scenarios, the locations of which are marked in Figure 6. Scene 1 was located on the old runway. Six golf balls with a diameter of 43 mm were placed around the co-ordinates (100 m, 300 m) and near the aircraft parked on the runway, three of which were in the same range bin. Scene 2 was located on the new runway, where seven metal balls with a diameter of 50 mm were placed around the co-ordinates (660 m, 250 m). Figure 7 shows the FOD targets used in the experiment.

## 5. Results and Discussion

### 5.1. Scene 1

**Figure 8:**
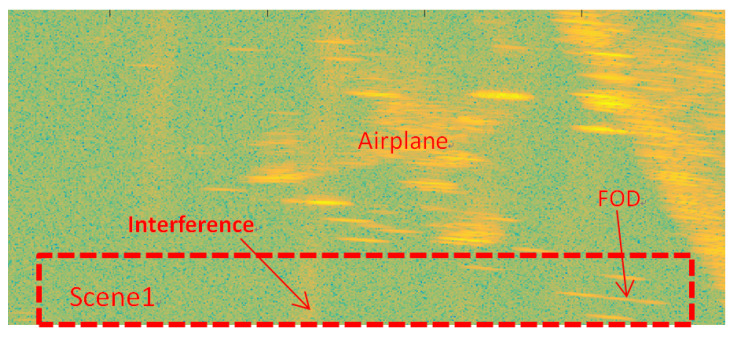
FOD targets deployment in Scene 1.

**Figure 9:**
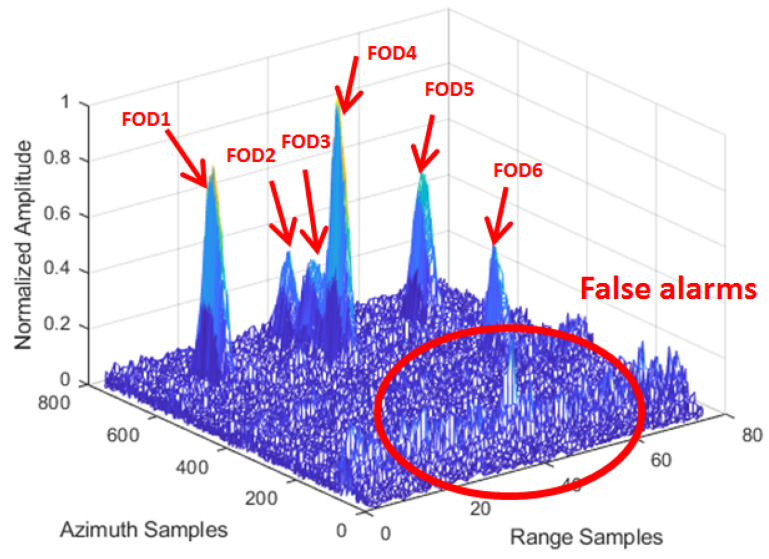
Before IAA processing of the area of Scene 1.

Figure 8 shows the amplitude image of the test Scene 1 after clutter cancellation processing. It can be seen that the strong echoes generated by airplane targets and grass could not be completely eliminated by clutter cancellation processing, with its side-lobes forming clutter interference resulting in false alarms, as shown in Figure 9. At the same time, there were many discrete clutter residues around the targets, which led to unnecessary false targets. Due to the poor azimuth resolution of the real aperture radar, it is obvious that the three close targets could not be distinguished.

**Figure 10:**
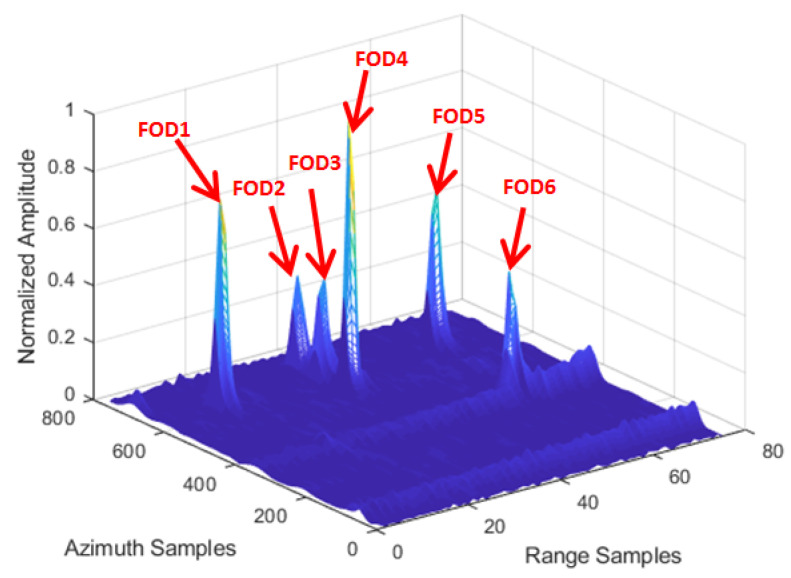
After IAA processing of the area of Scene 1.

**Figure 11:**
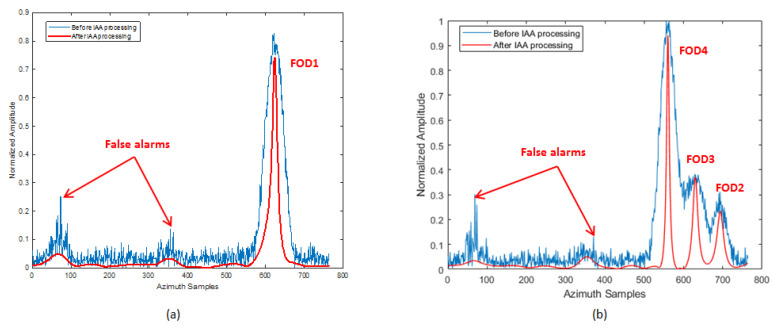
Comparison of IAA processing results for the same range bin: (**a**) before and after IAA processing of azimuth data on the 14th range bin of Scene 1; (**b**) before and after IAA processing of azimuth data on the 37th range bin of Scene 1.

According to the FOD Detection Method described in Section 3, the original data of the same range bin corresponding to the FOD target position were reprocessed by IAA in the azimuth direction for the refined data processing step. We used the known antenna reference pattern and azimuth echo data to reconstruct the signal based on IAA algorithm in the target area. Through IAA processing, it can be seen that the clutter and interference signals were suppressed, and signals overlapping in azimuth were also distinguished, as can be seen in Figure 10. In order to reveal the effect more clearly, Figure 11 shows the results of before and after the IAA processing of azimuth data in the same 14th and 37th range bins. It can be clearly seen, from the figure, that a group of interferences located near the 50th azimuth sampling point was suppressed. This was the side-lobe interference from the aircraft target. However, FOD 2 was retained and recovered, whose signal power was the same as the interference. At the same time, the single discrete interferences located near azimuth sampling point 371 were suppressed, as shown in Figure 11b, which were considered residual interference after the clutter cancellation. In summary, while the false alarms were eliminated, the clutter background was also simultaneously smoothed. The azimuth resolution of the targets was improved. The super-resolution performance is uniformly analyzed below.

**Figure 12:**
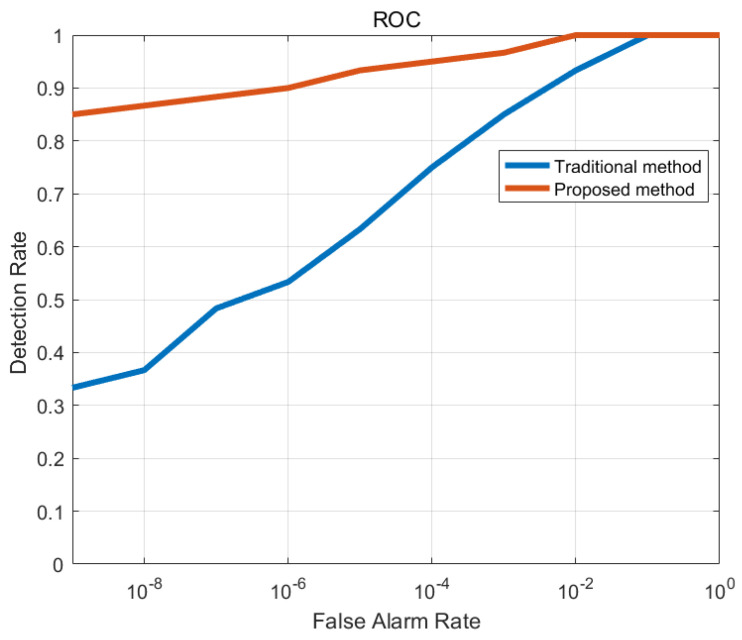
Comparison of ROC curves of the traditional and proposed detection methods.

After data processing based on IAA, CFAR detection processing was performed once more. The same cell averaging (CA) CFAR was used, with five guard cells in range and 50 guard cells in azimuth. Receiver operating characteristic (ROC; detection rate versus false alarm rate) analysis has been widely used as an evaluation tool for signal detection [37]. In order to evaluate the performance of the two methods, comparison of the ROC curves of the traditional and proposed detection method is shown in Figure 12. When the same false alarm probability parameter was set, the proposed method had higher detection probability. In other words, it provided a much lower false alarm rate. Meanwhile, the detection performance was more stable under different false alarm probabilities. In short, the results show that the detection performance of the method proposed in this article was significantly better than that of the traditional method.

### 5.2. Scene 2

**Figure 13:**
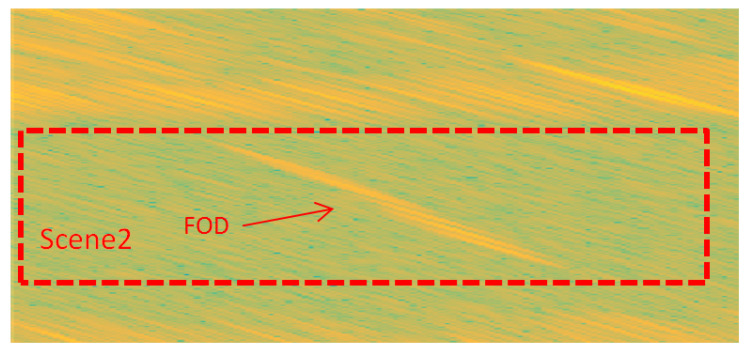
FOD target deployment, Scene 2.

Compared with the close range scene, the clutter was weaker in the long range test scene. There were almost no false alarms, but, due to the poor azimuth resolution of the radar, it could not be distinguished completely along the azimuth direction in Figure 13. The azimuth resolution of the real aperture scanning radar is related to the azimuth beam width of the antenna and the target distance; in particular, as the distance increases, the resolution decreases.

**Figure 14:**
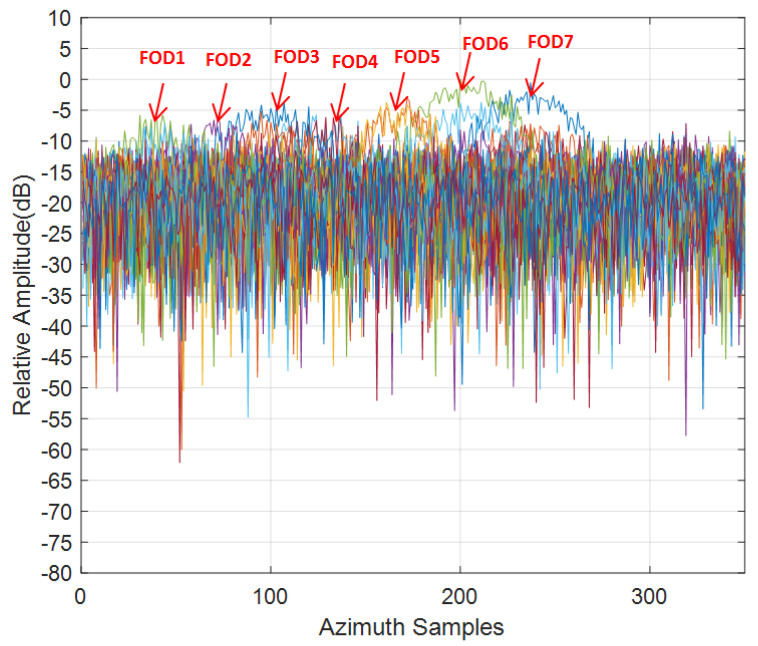
Test data obtained on azimuth direction, Scene 2.

**Figure 15:**
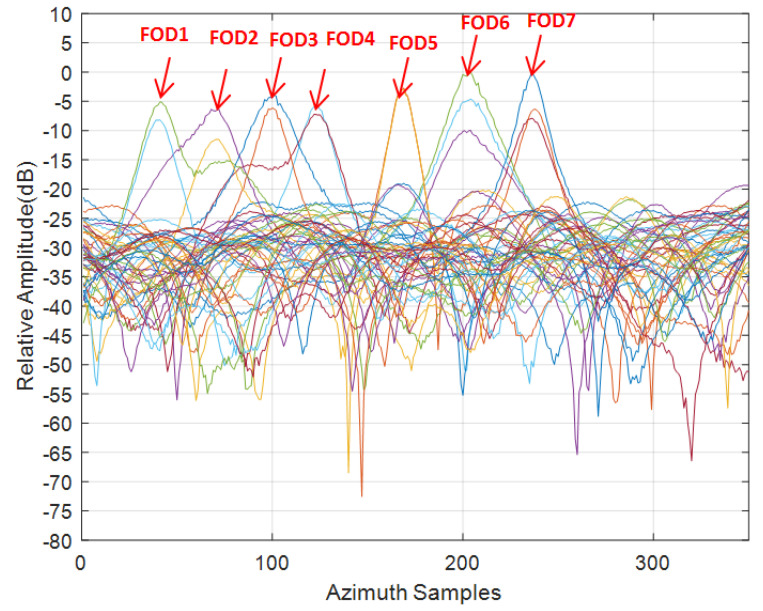
Results after azimuth IAA processing, Scene 2.

As the target was about 660 m away from the radar, the azimuth resolution was very poor. As can be seen from Figure 14, the overall clutter and noise level were relatively high, the SNR of the targets did not exceed 16 dB, and all targets were difficult to distinguish in the azimuth direction, leading to serious azimuth ambiguity. The IAA processing showed good azimuth super-resolution capability and suppression of the clutter background by about 10 dB, as shown in Figure 15. For FOD 5∼7 targets, as the signal-to-noise ratio was relatively higher, the reconstructed signal was smoother and the resolution effect was better, compared with the other FOD targets. To study the performance of super-resolution in this FOD detection method proposed, the super-resolution performance was evaluated using the super-resolution ratio, which is defined as [38]:

(19) $$\kappa =\frac{\Delta \theta }{\Delta S},$$

where Δθ is the main lobe width of the antenna pattern, which is equal to the azimuth resolution of the real beam imaging. ΔS had 3 dB width for the IAA estimates for a single FOD target. As determined through the calculation of each target in the scene, the super-resolution ratio κ statistics are listed in Table 2 and Table 3. The overall super-resolution performance of the FOD targets in Scene 1 was better than that of the FOD targets in Scene 2, where the FOD 4 target had the best super-resolution performance (of 3.1 times), benefiting from its high SNR. In Scene 2, the SNR was generally low (even down to 8 dB); however, a resolution performance of about 1.3 times was still obtained. These statistical results confirm that the IAA algorithm can maintain better performance at a lower SNR and, at the same time, as the SNR increases, the method’s performance increases [31,39].

Based on the analysis above, we have reason to believe that the method proposed in this paper not only possesses superior interference suppression capability, leading to false alarm reduction, but also can provide super-resolution in the azimuth direction.

## 6. Conclusions

In this article, a novel interference suppression and false alarm reduction method for a FOD millimeter-wave radar system was proposed, based on an iterative adaptive approach algorithm. The geometric and signal models of the real aperture FOD scanning radar were described. Next, the detection method based on IAA was derived in detail. Specifically, we first obtain coarse FOD target information by data preprocessing in a conventional detection method. Then, a refined data processing step is conducted based on the IAA algorithm in the azimuth direction. Finally, multiple pieces of information from the two steps above are used to comprehensively distinguish false alarms by fusion processing; thus, we can acquire accurate FOD target information. Real airport data measured by a 93 GHz radar are used to validate the proposed method. Experimental results of the test scene, which include golf balls with a diameter of 43 mm, were placed about 300 m away from radar, which show that the proposed method can effectively reduce the number of false alarms when compared with a traditional FOD detection method. Although metal balls with a diameter of 50 mm were placed about 660 m away from radar, they also can obtain up to 2.2 times azimuth super-resolution capability. Overall, the results demonstrated that the proposed method can effectively reduce false alarms and achieve super-resolution capability.

The IAA method has its limitations in some application scenarios, such as closely-spaced four targets and so on. How to improve and optimize its performance is an important work in the future. Recently, a new approach using Independent Component Analysis (ICA) with the Joint Approximate Diagonalization of Eigenmatrices (JADE) algorithm was used for separating closely-spaced subjects, such as respiratory signatures [40,41]. The research demonstrates that this approach can maintain accurate and efficient monitor multiple subjects across a broad range of subject separation scenarios [41]. Blind source separation (ICA-JADE) technology is a potential solution for a closely-spaced multiple targets separation in FOD detection. It may encourage further development toward a super-resolution FOD radar system.

## Figures and Tables

**Table 1:** Parameters of the FOD millimeter-wave radar system.

Parameter	Value	Units
Carrier frequency	93	GHz
Band width	2	GHz
Antenna scanning velocity	15	degrees/s
Azimuth main-lobe beamwidth	0.6	degree
Elevation main-lobe beamwidth	4	degrees
Antenna scanning area	−40∼+70	degrees
Pulse repetition frequency	1000	Hz

**Table 2:** Super-resolution ratio statistics of the FOD targets in Scene 1.

Target	FOD 1	FOD 2	FOD 3	FOD 4	FOD 5	FOD 6
SNR	16.2	10.1	12.2	18.3	13.8	12
κ	2.2	2.1	2.4	3.1	2.1	2.6

**Table 3:** Super-resolution ratio statistics of the FOD targets in Scene 2.

Target	FOD 1	FOD 2	FOD 3	FOD 4	FOD 5	FOD 6	FOD 7
SNR	9.8	8	10	8.2	12.3	15.2	14
κ	1.8	1.3	1.4	2	2.2	1.7	2.2

## Data Availability

Not applicable.
